# Significance of androgen receptor and its potential for anti-androgen/androgen receptor-antagonist therapy in ovarian cancers

**DOI:** 10.1371/journal.pone.0322744

**Published:** 2025-05-20

**Authors:** Teresa H. Kim, Sanaz Memarzadeh, Saba Vahdatshariatpanahi, Nora Ostrzega, Yuna Kang, Neda A. Moatamed

**Affiliations:** 1 Department of Pathology and Laboratory Medicine, David Geffen School of Medicine at UCLA, Los Angeles, CA,; 2 Department of Obstetrics and Gynecology, David Geffen School of Medicine at UCLA, Los Angeles, CA,; 3 UCLA Eli and Edythe Broad Center of Regenerative Medicine and Stem Cell Research, University of California Los Angeles, Los Angeles, CA,; 4 Johnson Comprehensive Cancer Center, University of California Los Angeles, Los Angeles, CA,; 5 Molecular Biology Institute, University of California Los Angeles, Los Angeles, CA,; 6 VA Greater Los Angeles Healthcare System, Los Angeles, CA.; Shiraz University of Medical Sciences, IRAN, ISLAMIC REPUBLIC OF

## Abstract

**Introduction:**

Androgen promotes tumorigenesis in some cancers; however, androgen receptor (AR) is not commonly examined in ovarian cancers (OCs). In this study, we evaluated AR expression among different types of OCs and compared the results to estrogen and progesterone receptors (ER & PR).

**Materials and methods:**

AR, ER, and PR expressions were assessed in 62 cases which were categorized into: low-grade serous carcinoma (LGSCA), high-grade serous carcinoma (HGSCA), clear cell carcinoma (CCCA), ovarian endometrioid carcinoma (OECA), and granulosa cell tumor (GCT). The hormone receptors were compared and evaluated in relation to p53 and body mass index (BMI) using Fisher’s Exact test.

**Results:**

In a majority of cases, expression of AR was concordant with ER and/or PR. Positivity for all three receptors was observed in 100% of OECAs. AR expression was seen in 92% of HGSCAs as opposed to 88% and 44% for ER and PR. LGSCAs had expressed AR and ER (100%), and PR (70%). In GCTs, positivity rates were 92%, 62%, and 92% for AR, ER, and PR. In rare cases of HGSCA and CCCA, AR was positive despite negative ER and PR.

**Conclusion:**

AR is expressed in a high percentage of OCs, even more frequently than ER and PR in certain high-grade histological types. Overall, our findings are similar to the results of recent studies of AR expression in endometrial cancers. These findings support an important possible role for AR in OCs as a potential marker to serve as a therapeutic target in these malignancies.

## Introduction

Due to a variety of cellular origins, ovarian cancers (OC) represent the categories of heterogeneous diseases that include germ cell, sex-cord/stromal, and epithelial tumors [1]. Each category is further subdivided by histological types that also differ in etiology, grade, therapeutic response, and prognosis. Sex-cord/stromal tumor categories develop from cells that support the ovary by producing hormones such as estrogen and progesterone, where granulosa cell tumor (GCT) is a rare malignant entity that falls under this category [2]. More than 85% of OCs are believed to develop from ovarian surface epithelial cells, and these tumors are some of the most lethal gynecologic malignancies, causing OC to be the fifth leading cause of cancer-related deaths in women [3,4]. Ovarian carcinomas are further subdivided into several major histological subtypes: serous, endometrioid, clear cell, and mucinous [1].

Steroid hormones have been implicated in the development of epithelial ovarian cancers. Fundamentally, androgen action is an important part of normal reproductive function in the ovary [5]. In experimental studies, androgen receptor (AR) expression has been detected in several cell types, including somatic cells during different stages of life [6–8]. In addition, androgen/AR signaling has been observed to promote tumorigenesis in different cancer types, including OC [9]. Further investigation has demonstrated that androgen stimulation promotes cancer progression through regulation of the cell cycle in AR-positive serous carcinomas [10]. These findings, in addition to other studies [2,10,11], support an important potential role for androgen/AR in OCs as a possible marker to serve as a therapeutic target in these malignancies. However, the results of several clinical trials evaluating the effects of anti-androgen drugs on OC have been less encouraging, and therefore, the exact function of AR in this disease model remains unknown [12–15].

Current therapeutic strategies in treating OC are based on the stage and grade at diagnosis and consist of surgical bulk reduction followed by adjuvant chemotherapy [16,17]. However, advanced-stage patients have lower survival and higher relapse rates. Several neoplasms of the female genital tract are associated with hormone exposure and benefit from hormonal therapy targeting estrogen (ER) and progesterone (PR) receptors [18]. Although androgens are known precursors of estrogen, there are limited studies investigating the effects of hormonal therapy targeting AR in OC. The role of AR in other non-gynecologic neoplasms, such as prostate cancer, is well-established and commonly treated with androgen antagonists [19]. When the ligand binds to AR, the receptor dissociates from the accessory proteins and translocate into the nucleus, where it subsequently binds to androgen response element in the promoter region of the gene, resulting in cellular proliferation and apoptosis avoidance [20–22]. Up to 90% of prostate cancers are androgen dependent at the time of initial diagnosis. Although androgen deprivation is an effective treatment, 20–30% of the cancers become resistant [23], paradoxically allowing the disease to progress through alternative signaling pathways, including AR mutations, splice variants, and increased production of growth factors [24].

Additionally, the use of AR-targeted treatment in certain types of breast cancer is an ongoing area of research [25–27].

Initial studies, before recent WHO classification of tumors, had demonstrated overexpression of AR in OCs [28–30]. Of note, AR has been found to be the most abundant of the steroid receptors in GCT [31]. More recent studies have also shown AR expression in nearly all OC subtypes, with higher levels seen in serous carcinomas [32,33]. The goal of this study was to investigate AR expression among different types of OCs and stratify our results, as well as those of others, using the ovarian cancers diagnostic categories according to the recent WHO classification of GYN tumors. In addition, we compared the AR results to ER, PR, p53, body mass index (BMI), and tumor recurrence as a measure of resistance to the chemotherapy in OCs, as a sequel to our study in endometrial cancers [34]. p53 and BMI are of interest in this study setting. TP53 mutation is almost invariably present in Müllerian high grade serous carcinomas [35]. Association of BMI in the female cancers is captured in a meta-analysis study, where the results have shown an increased risk of ovarian serous and endometrioid cancers in patients with high BMIs [36–38].

## Materials and methods

The current retrospective study was approved by the Institutional Review Board (IRB #22–000672) at UCLA David Geffen School of Medicine. Due to the retrospective nature of this study which used archived information and tissue blocks, no patients were involved and the need for patient’s consent had been waived by the UCLA IRB. A search for ovarian cancers (OCs) diagnosed between April 2016 to February 2023 was conducted through the Epic Beaker laboratory information system. The inclusion criteria for the search contained cases of OCs who had reported results of immunohistochemical (IHC) stains for ER and PR, performed for patient care. The results of other tests, such as mismatch repair (MMR), Her-2neu, and p53, were obtained if also available and had been ordered during routine pathology practice. In addition, some patient demographic information, such as age, BMI, months of survival following the initial diagnosis, and treatment was recorded. The nomenclature and FIGO (International Federation of Gynecology and Obstetrics) grades for all primary ovarian malignancies were documented according to the most current established WHO guidelines [39].

### IHC stains

All materials and staining steps for AR, ER, PR, mismatch repair (MMR) proteins, and p53 were carried out exactly according to the previously published protocols [34]. For AR, prostate tissue is universally used as control for the IHC stain as it was in our study [40]. When present in the specimen, normal ovarian tissue was also used as internal control (S1 Fig). Normally, AR is expressed in normal ovarian tissue including oocytes, granulosa cells, and theca cells [41]. The IHCs for ER, PR, and p53 had previously been performed on these cases for diagnostic or clinical purposes, and the data were recorded as available in the patients’ clinical records. Only AR IHC was performed on the same tissue sections for this study.

### IHC stain scoring

Scoring for ER and PR had been performed according to the current College of American Pathologists (CAP)/American Society of Clinical Oncology (ASCO) guidelines for breast cancer, in which any cellular reaction of 1% or more was considered a positive result [42,43]. An identical procedure was adopted for scoring AR IHC in this study as previously described for endometrial cancers [34]. Weak, moderate, and strong nuclear staining was recorded as 1 + , 2 + , and 3 + intensities, respectively. Both the percentage of positive cancer cells as well as the intensity of the stains were recorded for probable future meta-analysis. All the stains were carried out on the whole tissue sections, using no tissue micro-arrays. Two of the authors re-reviewed the stains and any discrepant cases were further double scoped by two other pathologists as in the previous study [34]. Subsequently, we recorded the final stain interpretations for this series.

### p53

p53 protein tends to accumulate in the nuclei of the cancer cells with TP53 missense mutations. In normal tissues, wild type (WT) staining represents the scattered nuclear IHC reaction which is present in less than 30% of the cells. When the mutation occurs, there are two general IHC patterns of the aberrant expression (AE): one is diffuse and strong nuclear and sometimes cytoplasmic staining of more than 80% of the cells while the other is complete absence of the reaction [44]. The AEs were interpreted as positive and WT as negative.

### Body mass index (BMI)

We followed the used WHO guidelines for defining body mass indices (kg/m^2^) as follows: underweight (BMI < 18.5), normal or desirable weight (BMI = 18.5 to < 25), over-weight (BMI = ≥25 to < 30), and obese (BMI ≥ 30) [17]. In this study, we considered BMIs < 25 (normal + underweight) as negative and BMIs ≥ 25 (Overweight + obese) as positive and used this to compare to the receptors for concordance as we did for the patients with endometrial cancers [34].

### Chemotherapy response

Recurrence (RR) of the tumor was used as an indicator of resistance to the chemotherapy, Elevation of CA125 level in serum [45,46], imaging [47], elevated serum level of Inhibin [48–50], and/or combination of positive cytology and biopsy became the indicators of recurrence.

### Statistical analysis

Fisher’s Exact test in a 2x2 contingency table [51], was utilized to compare the AR, ER, and PR IHC stains to each other, in addition to comparing them to p53, BMI, and recurrence. Other tests, including Her2/neu, mismatch repair (MMR) proteins, and FOXL2 mutation tests had been ordered on a few patients for which the test results were recorded (S1 Table) but not included in the statistical analysis due to the paucity of the numbers. To reject the null hypothesis, *P*-values of .05 or less were used to indicate statistically significant differences between the two data sets. *P-*values of greater than .05 but less than 0.1 were considered as borderline significance.

### Study design

The results of androgen, estrogen, and progesterone receptor staining were tabulated based on five diagnostic categories: (low grade serous carcinoma (LGSCA), high grade serous carcinoma (HGSCA), clear cell carcinoma (CCCA), ovarian endometrioid carcinoma (OEC), and granulosa cell tumor (GCT). Each steroid hormone receptor was interpreted as positive when the nuclear staining was present in 1% or more of the cancer cells according to the guidelines set by the College of American Pathologists/American Society of Clinical Oncology [42,43]. The estimated percentage of cellular positivity and intensity of staining were all tabulated (S1 Table). Fisher’s Exact test in 2x2 contingency tables, using positive and negative results in the five diagnostic categories, was used to compare the three receptor stains to each other and also to p53, BMI, and RR. For comparing to p53 only the cases with p53 test results and for RR those patients who had received chemotherapy were selected for the statistical analyses. The study design and the statistical analyses were carried out similar to that in the previous study [34].

We adhered to aspects of the REMARK guidelines which were relevant to this study in designing and conducting this work [52]. Since androgen antagonists or anti-AR agents were not used on the patients in a clinical trial, the survival or outcome part of the guidelines did not pertain to the current series [52].

## Results

Out of the 399 cases of ovarian cancer obtained during the search period, the system returned 62 surgical pathology cases matching the inclusion criteria. For this study, AR staining was performed and evaluated on these 62 cases. Patients’ ages ranged from 18 to 85 years with a median of 61. Forty-three cases had chemotherapy, among which 9 had received additional anti-estrogen (aromatase inhibitor) treatment (S1 Table). Of the total, 11 patients had received no treatment and 8 were lost to the follow-up. None of the cases had received treatment with anti-androgen/androgen receptor-antagonist agents. Six patients had expired at the conclusion of this study on November 27, 2023 (S1 Table). The number of patients with AR positivity and their pertinent clinical status, including recurrences, are summarized in Table 1.

**Table 1:** Androgen receptor positivity and status of the pertinent clinical features of the patients for each diagnostic category.

Dx	n	AR+	Tumor Stage			Chemo Rx	Hrmn Rx	NF	No Rx	RR^¶^	Alive^*^	Dead
			1	2	3							
**LGSCA**	10	**10**	**4**	0	**6**	**6**	**3**	**3**	**1**	**5**	**8**	**2**
**HGSCA**	25	**23**	**4**	**4**	**17**	**23**	0	**2**	0	**12**	**22**	**3**
**CCCA**	6	**1**	**2**	0	**4**	**5**	0	**1**	0	**3**	**5**	**1**
**OECA**	8	**8**	**5**	**2**	**1**	**3**	0	**1**	**4**	**1**	**8**	0
**GCT**	13	**12**	**10**	**1**	**1**	**6**	**6**	**1**	**6**	**5**	**13**	0
**All**	62	**54** (87%)	**25** (40%)	**7** (11%)	**29** (47%)	**43** (69%)	**9** (15%)	**8** (13%)	**11** (18%)	**26** (48%)	**56** (90%)	**6** (10%)

Dx, diagnosis; AR + , androgen receptor, positive; RX, therapy; Hrmn, hormonal; NF, no follow up; RR, recurrence; LGSCA, low grade serous carcinoma; HGSCA, high grade serous carcinoma; CCCA, clear cell carcinoma; OECA, ovarian endometrioid carcinoma; GCT, granulosa cell tumor.

¶ NF cases excluded in calculations.

^*^As of conclusion of the study.

The cancer cell positivity ranged from 1% to 100% for the three receptors using the IHC stains (S1 Table). Overall, the rate of positivity was 77% (48/62) for ER, 61% (38/62) for PR, and 87% (54/62) for AR (Table 2). In addition, combined 2 + & 3 + intensities were observed in 60% (37/62) of ER, 53% (33/62) of PR, and 84% (52/62) of AR reactions as listed in S1 Table. These findings indicate higher rates of positivity and stronger intensity (expression) of the AR.

**Table 2:** Summary of data including the immunostains results and the statistical tests’ *P*-values in the ovarian cancers.

Diagnosis	Age	n	ER		PR		AR	
			+n	+%	+n	+%	+n	+%
**LGSCA**	62.55	10	10	**100%**	7	**70%**	10	**100%**
**HGSCA**	66	25	22	**88%**	11	**44%**	23	**92%**
**CCCA**	53.5	6	0	0%	0	0%	1	**17%**
**OECA**	56	8	8	**100%**	8	**100%**	8	**100%**
**GCT**	61	13	8	**62%**	12	**92%**	12	**92%**
**All**	61	62	48	**77%**	38	**61%**	54	**87%**
**2x2 Contingency-Table Values**						**Fisher’s Exact-Test *P*-Values**		
		**Positive**	**Negative**			**ER** vs **PR**	**ER** vs **AR**	**PR** vs **AR**
**LGSCA**	**ER**	**10**	0					0.2
	**PR**	**7**	**3**				1*	
	**AR**	**10**	0			0.2		
**HGSCA**	**ER**	**22**	**3**					**0.0006** ^**§**^
	**PR**	**11**	**14**				1	
	**AR**	**23**	**2**			**0.002** ^**§**^		
**CCCA**	**ER**	0	**6**					1
	**PR**	0	**6**				1	
	**AR**	**1**	**5**			1*		
**OECA**	**ER**	**8**	0					1*
	**PR**	**8**	0				1*	
	**AR**	**8**	0			1*		
**GCT**	**ER**	**8**	**5**					1
	**PR**	**12**	**1**				0.2	
	**AR**	**12**	**1**			0.2		
**All**	**ER**	**48**	**14**					**0.002** ^**§**^
	**PR**	**38**	**24**				0.2	
	**AR**	**54**	**8**			**0.08** ^**#**^		

**Age**, median; **ER**, estrogen receptor; **PR**, progesterone receptor; **AR**, androgen receptor; **LGSCA**, low grade serous carcinoma; **HGSCA**, high grade serous carcinoma; **CCCA**, clear cell carcinoma; **OECA**, ovarian endometrioid carcinoma; **GCT,** granulosa cell tumor; **+ **, positive

§ Significant *P*-values.

^**#**^Borderline *P*-value.

*Assumed since both sets of data are identical and the statistical application did not return results due to the two zeros in one column.

When the three stains were compared against each other in the combined five diagnostic categories (LGSCA, HGSCA, CCCA, OECA, and GCT), Fisher Exact tests returned a significant *p*-values for AR versus PR (*p*-value = 0.002) and a borderline value (*p*-value = 0.08) for ER versus PR. There was no significant difference between AR and ER (*p*-value = 0.2). The results indicated that the lower rate of PR expression was responsible for the significant differences while the difference between ER and AR remained insignificant (Table 2).

Among these 62 cases, 44 (71%) had p53 IHC tests performed for patient care purposes. The cases with no p53 were excluded from the statistical test. There were 57% (25/44) of patients who had aberrant expression of p53 (S1 Table) only observed in high grade serous carcinoma (Table 3). The receptors’ stain results were individually compared to p53 in all 44 patients. Fisher’s Exact test showed a significant difference when p53 was compared to AR (*p*-value = 0.004), where p53 had an aberrant expression in 57% (25/44) whereas AR had been expressed in 86% (38/44) of the cases. The comparative differences between p53 and ER was also significant (*p*-value = 0.009), where PR’s difference with p53 remained insignificant (*p*-value = 0.8) (Table 3).

**Table 3:** Summary of data including the immunostains results of the receptors and p53 plus the related statistical tests’ *p*-values for the cases having immunohistochemical stains for all fours markers in the ovarian cancers.

Diagnosis	n	ER		PR		AR		p53	
		+n	+%	+n	+%	+n	+%	+n	+%
**LGSCA**	10	10	**100%**	7	**70%**	10	**100%**	0	**0%**
**HGSCA**	25	22	**88%**	11	**44%**	23	**92%**	25	**100%**
**CCCA**	4	0	**0%**	0	**0%**	0	**0%**	0	**0%**
**OECA**	5	5	**100%**	5	**100%**	5	**100%**	0	**0%**
**GCT**	0	0	**0%**	0	**0%**	0	**0%**	0	**0%**
**All**	44	37	**84%**	23		38	**86%**	25	**57%**
**2x2 Contingency-Table Values**						**Fisher’s Exact-Test *P*-values**			
		**Positive**	**Negative**				**ER** vs **p53**	**PR** vs **p53**	**AR** vs **p53**
**LGSCA**	**ER**	10	0					**0.003** ^**§**^	
	**PR**	7	3				**0.00001** ^**§**^		**0.00001** ^**§**^
	**AR**	10	0						
	**p53**	0	10						
**HGSCA**	**ER**	22	3					**0.00001** ^**§**^	
	**PR**	11	14				0.2		0.5
	**AR**	23	2						
	**p53**	25	0						
**CCCA**	**ER**	0	4					1*	
	**PR**	0	4				1*		1*
	**AR**	0	4						
	**p53**	0	4						
**OECA**	**ER**	5	0					**0.008** ^**§**^	
	**PR**	5	0				**0.008** ^**§**^		**0.008** ^**§**^
	**AR**	5	0						
	**p53**	0	5						
**All**	**ER**	37	7					0.8	
	**PR**	23	21				**0.009** ^**§**^		**0.004** ^**§**^
	**AR**	38	6						
	**p53**	25	19		**52%**				

ER, estrogen receptor; PR, progesterone receptor; AR, androgen receptor; LGSCA, low grade serous carcinoma; HGSCA, high grade serous carcinoma; CCCA, clear cell carcinoma; OECA, ovaria endometrioid carcinoma.

+ positive.

§ Significant *P*-values; GCT, granulosa cell tumors were excluded for statistical analysis since none of them had the associated p53 test.

*Assumed since both sets of data are identical and the statistical application did not return results due to the two zeros in one column.

BMIs of 25 (kg/m^2^) or greater were observed in 45% (28/62) of the total patients, among which 11% (7/62) had a BMI of 30 or more (S1 Table) falling in the obese classes [17]. Fisher’s Exact test returned significant *p*-values for ER (p-value = 0.0004) and AR (p-value < 0.0001) when the receptor stains were compared to BMIs of ≥ 25 in the “All” category, indicating lack of concordance of the BMIs with the receptor stains unlike PR (p-value = 0.1) (Table 4).

**Table 4:** Summary of the data including the immunostain test results and the statistical tests’ *p*-values as the receptors were compared to BMI (body mass indices) in the ovarian cancers.

Diagnosis		n	ER		PR		AR			BMI
			+n	+%	+n	+%	+n	+%	+n	+%
**LGSCA**		10	10	**100%**	7	**70%**	10	**100%**	3	**30%**
**HGSCA**		25	22	**88%**	11	**44%**	23	**92%**	10	**40%**
**CCCA**		6	0	0%	0	0%	1	**17%**	3	**50%**
**OECA**		8	8	**100%**	8	**100%**	8	**100%**	4	**50%**
**GCT**		13	8	**62%**	12	**92%**	12	**92%**	8	**62%**
**All**		62	48	**77%**	38	**61%**	54	**87%**	28	**45%**
**2x2 Contingency-Table Values**						**Fisher’s Exact-Test *P*-Values**				
		**Positive**	**Negative**			**ER** vs **BMI**	**PR** vs **BMI**		**AR** vs **BMI**	
**LGSCA**	**ER**	**10**	**0**				0.2			
	**PR**	**7**	**3**			**0.003** ^**§**^			**0.003** ^**§**^	
	**AR**	**10**	**0**							
	**BMI**	**3**	**7**							
**HGSCA**	**ER**	**22**	**3**				1			
	**PR**	**11**	**14**			**0.0009** ^**§**^			**0.0002** ^**§**^	
	**AR**	**23**	**2**							
	**BMI**	**10**	**15**							
**CCCA**	**ER**	**0**	**6**				0.2			
	**PR**	**0**	**6**			0.2			0.5	
	**AR**	**1**	**5**							
	**BMI**	**3**	**3**							
**OECA**	**ER**	**8**	**0**				**0.08** ^**#**^			
	**PR**	**8**	**0**			**0.08** ^**#**^			**0.08** ^**#**^	
	**AR**	**8**	**0**							
	**BMI**	**4**	**4**							
**GCT**	**ER**	**8**	**5**				0.2			
	**PR**	**12**	**1**			1			0.2	
	**AR**	**12**	**1**							
	**BMI**	**8**	**5**							
**All**	**ER**	**48**	**14**				0.1			
	**PR**	**38**	**24**			**0.0004** ^**§**^			**<0.0001** ^**§**^	
	**AR**	**54**	**8**							
	**BMI**	**28**	**34**							

**ER**, estrogen receptor; **PR**, progesterone receptor; **AR**, androgen receptor; **LGSCA**, low grade serous carcinoma; **HGSCA**, high grade serous carcinoma; **CCCA**, clear cell carcinoma; **OECA**, ovarian endometrioid carcinoma; **GCT**, granulosa cell tumor.

^**+**^Positive; **BMI**, body mass index (Positive if BMI ≥ 25).

^§^Significant *p*-values; **#**, borderline *p*-values.

There were 7 cases (11% of the total) which had MMR IHC tests performed on the tumors for patient care purposes, showing intact genes in all 7 patients (S1 Table). Her2/neu was tested in 3 cases (5% of the total) with negative results (S1 Table). Also, 1 patient was tested for FOXL2 which was mutated (S1 Table). Due to the paucity of tests, MMR, Her2/neu, and FOXL2 results were not included in the statistical analyses.

Due to the discovery nature of this study, *p*-values were not corrected for multiple comparisons, and their nominal values should be interpreted cautiously as in the previous study [34].

Of the 43 patients who had received chemotherapy, among whom 60% (26/43) had recurrence at the conclusion of this study where 86% (37/43) and 81% (35/43) of their tumors were strongly positive for AR and ER respectively. Progesterone had the lowest positive reaction by IHC (53%). Fisher’s Exact test yielded a significant *p*-value of 0.01 when the AR positivity, in all diagnostic categories, was compared to the recurrent ovarian cancers (Table 5), meaning AR had been expressed in the cancers beyond those with recurrence.

**Table 5:** Summary of data including the immunostains results of the receptors and clinical recurrence (RR) in patients with chemotherapy and follow-ups plus the related statistical tests’ *p*-values for the cases having immunohistochemical stains for the three markers.

Diagnosis	n	ER		PR		AR		Chemo Rx Response	
		n+	+%	n+	+%	n+	+%	RR	RR%
**LGSCA**	6	6	**100%**	3	**50%**	6	**100%**	5	**83%**
**HGSCA**	23	20	**87%**	11	**48%**	21	**91%**	12	**52%**
**CCCA**	5	0	**0%**	0	**0%**	1	**20%**	3	**60%**
**OECA**	3	3	**100%**	3	**100%**	3	**100%**	1	**33%**
**GCT**	6	6	**100%**	6	**100%**	6	**100%**	5	**83%**
**All**	43	35	**81%**	23	**53%**	37	**86%**	26	**60%**
**2x2 Contingency-Table Values**								**Fisher’s Exact-Test *P*-values**	
		**Positive**	**Negative**				**ER** vs **RR**	**PR** vs **RR**	**AR** vs **RR**
**LGSCA**	**ER**	**6**	**0**					0.5	
	**PR**	**3**	**3**				1		1
	**AR**	**6**	**0**						
	**RR**	**5**	**1**						
**HGSCA**	**ER**	**20**	**3**					1	
	**PR**	**11**	**12**				**0.02** ^**§**^		**0.007** ^**§**^
	**AR**	**21**	**2**						
	**RR**	**12**	**11**						
**CCCA**	**ER**	**0**	**5**					0.2	
	**PR**	**0**	**5**				0.2		0.5
	**AR**	**1**	**4**						
	**RR**	**3**	**2**						
**OECA**	**ER**	**3**	**0**					0.4	
	**PR**	**3**	**0**				0.4		0.4
	**AR**	**3**	**0**						
	**RR**	**1**	**2**						
**GCT**	**ER**	**6**	**0**					1	
	**PR**	**6**	**0**				1		1
	**AR**	**6**	**0**						
	**RR**	**5**	**1**						
**All**	**ER**	**35**	**8**					0.7	
	**PR**	**23**	**20**				**0.06** ^**#**^		**0.01** ^**§**^
	**AR**	**37**	**6**						
	**RR**	**26**	**17**						

**ER**, estrogen receptor; **PR**, progesterone receptor; **AR**, androgen receptor; **Rx**, therapy; **RR**, recurrence (positive), no-recurrence (negative); **LGSCA**, low grade serous carcinoma; **HGSCA**, high grade serous carcinoma; **CCCA**, clear cell carcinoma; **OECA**, ovaria endometrioid carcinoma; **GCT**, granulosa cell tumor.

^**+**^** **Positive.

^**§**^Significant p-values.

^**#**^Borderline p-value.

### LGSCA (low grade serous carcinoma)

There were 10 cases in this diagnostic category. Patients’ ages ranged between 42–72 with a median of 62.5 years (Table 2). Six patients had chemotherapy, among which three had received additional anti-estrogen (anastrozole) treatment. Five of the 6 patients (83%) had recurrence where AR was positive in all 6 cases (Table 5). None of the patients had been treated with anti-androgen agents. Two cases had expired and eight were alive at the time of this study.

Three did not have their follow up at our institution (S1 Table).

**Fig 1:**
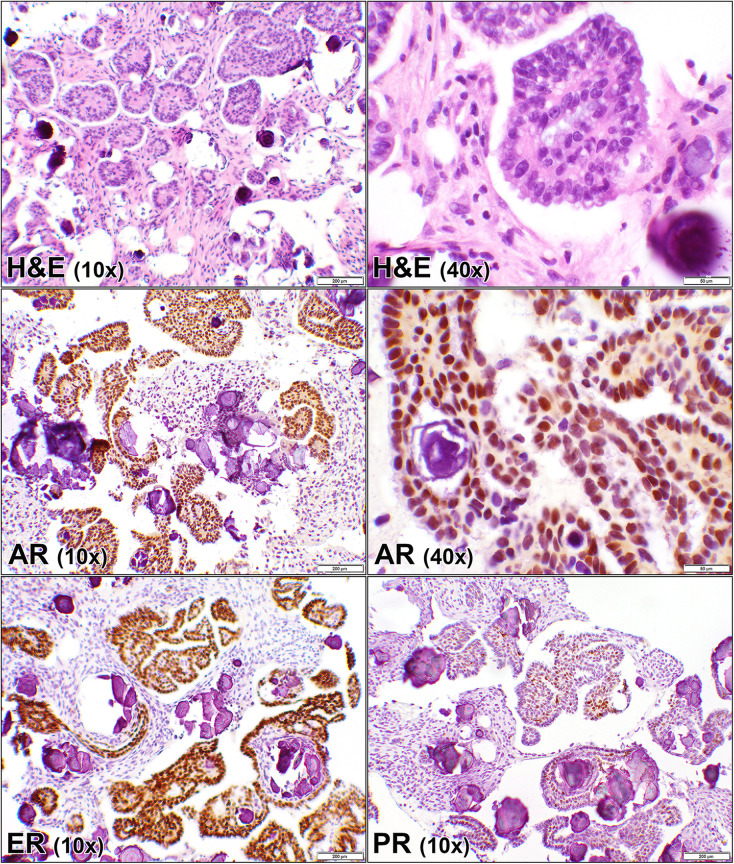
Androgen, estrogen, and progesterone receptors in ovarian low grade serous carcinoma. Hematoxylin and eosin (H&E) stain of a case (case #5, S1 Table) of an ovarian tumor showing glandular proliferation, composed of micropapillary pattern of invasion within clear spaces. Psammoma bodies are also present. The corresponding androgen receptor (AR), estrogen receptor (ER), and progesterone receptor (PR) immunostains show similar positive nuclear staining of the malignant cells although a weaker reaction for PR. (10 × and 40x objective).

AR and ER were positive in 100% (10/10) while PR stain was positive in 70% (7/10) of the patients as shown in Table 2 and represented in Fig 1. Combined 2 + & 3 + intensities of the respective reactions were observed in 100% (10/10) of AR and ER and 60% (6/10) for PR. Three of LGSCA (case #4, 6, 8, S1 Table) were negative for the PR. Overall, the cellular positivity ranged from 1% to 100% with the intensity of 1 + to 3 + for all three receptors (S1 Table). Since the positivity of the cells were similar for the three receptors, Fisher’s Exact test returned *p*-values of ≥ 0.2 when the stains were com*p*ared to each other in this diagnostic category indicating insignificant differences. Particularly, the *p*-value of AR versus ER was 1 (Table 2), all indicating concordances between the three rece*p*tors’ stains in this diagnostic category.

All specimens, in this category, had been tested with p53 immunostain and all showed a wild-type pattern of staining (S1 Table). p53 was significantly different when compared to each receptor using Fisher’s Exact test (*p*-values of ≤ 0.003) indicating the aberrant p53 expressions are lacking and do not correlate with the receptors’ positive expression (Table 3).

Among the 10 patients, 3% (3/10) were overweight (BMI ≥ 25) of which 20% (2/10) were obese (BMI ≥ 30) in this category. Comparison of BMI of ≥ 25 with the three receptors revealed a *p*-value of 0.2 for PR as opposed to the *p*-values of 0.003 for ER and AR indicating lack of concordance between being overweight versus positive expression of AR and ER (Table 4).

### HGSCA (high grade serous carcinoma)

There were 25 cases in this diagnostic category. Patients’ ages ranged between 35–85 with a median of 66 years (Table 2). Twenty-three patients had chemotherapy, among which none had received additional anti-estrogen (anastrozole) treatment or anti-androgen agents. Twelve of the 23 patients (52%) had recurrence where AR was positive in 91% (21/23) of the cases and Fisher’s Exact test returned a *p*-value of 0.007 (Table 5). Three patients had expired when this study was concluded on November 27, 2023 (S1 Table).

**Fig 2:**
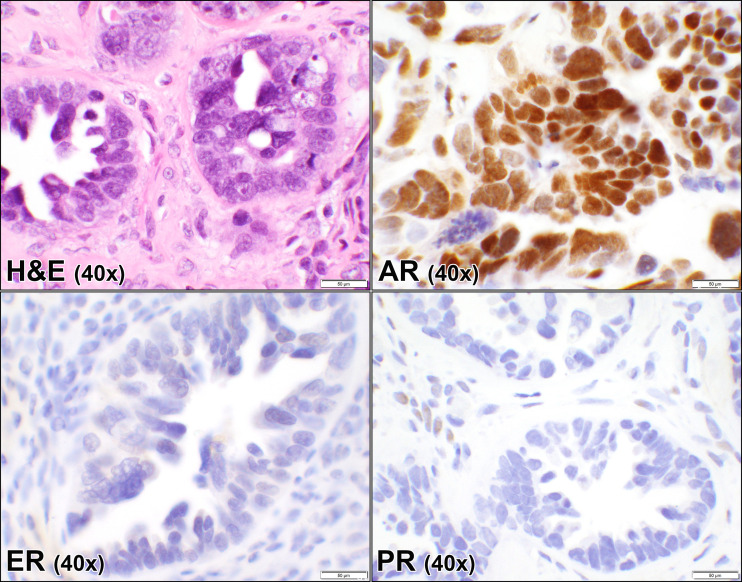
Androgen, estrogen, and progesterone receptors in ovarian high grade serous carcinoma. Hematoxylin and eosin (H&E) stain of a case of ovarian serous carcinoma showing glandular proliferation with marked nuclear pleomorphism and prominent nucleoli (case #40, S1 Table). The corresponding androgen receptor (AR), estrogen receptor (ER), and progesterone receptor (PR) immunostains show strong positive nuclear staining with AR and negative nuclear staining of the malignant cells for ER and PR. (40 × objective).

ER was positive in 88% (22/25), PR in 44% (11/25), and AR in 92% (23/25) of the cancers in this category as shown in Table 2 and represented in Fig 2. Overall, the cellular positivity ranged from 5% to 95% with combined 2 + & 3 + intensities in 68% (17/25), 32% (8/25), and 84% (21/25) for ER, PR, and AR respectively (S1 Table), indicative of the higher rate and stronger expression of AR in HGSCA diagnostic cancer category. Fisher’s Exact test returned significant *p*-values (≤0.002) for ER versus PR and AR versus PR, whereas an insignificant difference (*p*-value = 1) was observed when AR was com*p*ared to ER (Table 2).

The receptors’ expressions were insignificantly different (*p*-values ≥ 0.2) when AR and ER were compared to p53 aberrant expressions, while a similar comparison was significantly (*p*-value = 0.00001) different with PR (Table 3).

Among the 25 patients, 40% (10/25) were overweight (BMI ≥ 25) of which 8% (2/25) were obese (BMI ≥ 30) in this category. When the receptors’ expression rates were compared to the BMI (≥ 25), the results were variable. AR and ER versus BMI showed significant differences (*p*-values ≤ 0.0009), whereas PR versus BMI had a *p*-value of 1, namely PR was concordant while ER and AP were not with the patients being overweight (Table 4).

### CCCA (clear cell carcinoma)

There were 6 cases in this diagnostic category. Patients’ ages ranged between 44–73 with a median age of 53.5 years (Table 2). Five cases had received chemotherapy among which none had received additional anti-estrogen (anastrozole) or anti-androgen agents. Three of the 5 patients (60%) had recurrence where AR was positive in only 1 case (Table 5). At the conclusion of this study, one patient had expired and one was lost to the follow-up (S1 Table).

**Fig 3:**
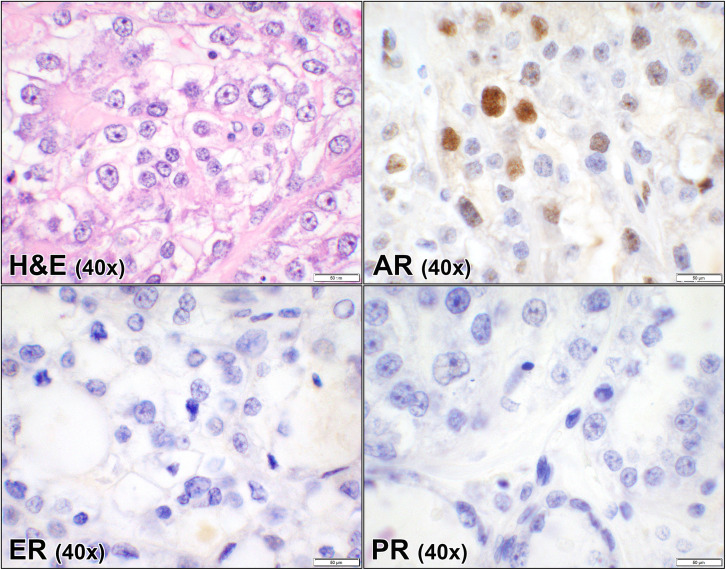
Androgen, estrogen, and progesterone receptors in ovarian clear cell carcinoma. Hematoxylin and eosin (H&E) stain of a case of clear cell carcinoma of the ovary showing malignant epithelial cells with clear cell cytoplasm and pleomorphic nuclei (case #39, S1 Table). The corresponding AR shows sparse focal nuclear staining while ER and PR are negative. (40x objective).

All 6 (100%) cases of CCCA were negative for ER and PR staining whereas one (17%) had expressed AR in which 20% of the tumor cells showed 2 + positivity. These results are summarized in Table 2 and represented in Fig 3.

p53 stain showed wild type reactions in 4/6 cases and was not performed in the remaining two cases. The comparison tests returned *p*-values of 1, showing a perfect correlation (Table 3).

Three patients were overweight (BMI ≥ 25) and when compared to the three receptors, the results were not significantly (p-values ≥ 0.2) different (Table 4).

### OECA (ovarian endometrioid carcinoma)

There were 8 cases in this diagnostic category. Patients’ ages ranged between 44–73 with a median of 56 years (Table 2). Three patients had chemotherapy while none had received anti-androgen agents, and all 8 cases were of low-grade carcinomas (FIGO grade 1 or 2) (S1 Table). One of the 3 patients (33%) had recurrence where AR was positive in all 3 cases (Table 5).

**Fig 4:**
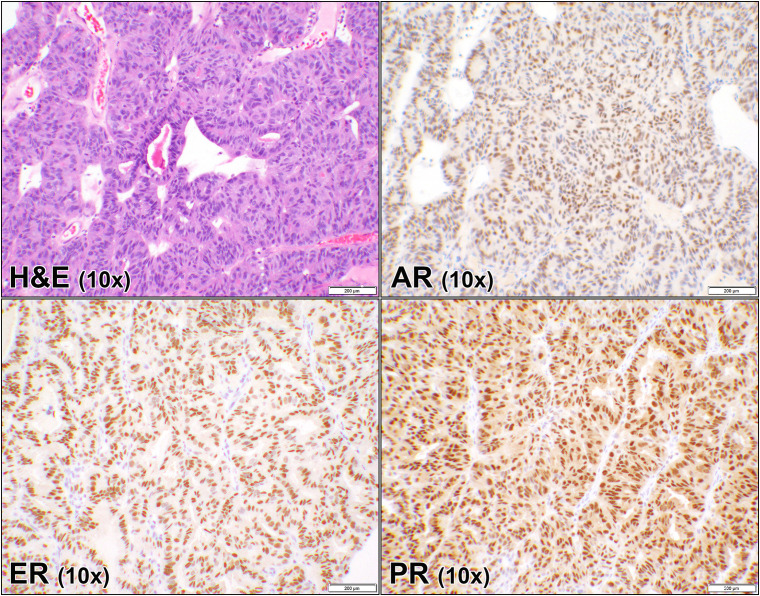
Androgen, estrogen, and progesterone receptors in ovarian endometrioid carcinoma. Hematoxylin and eosin (H&E) stain of a case (case #48, S1 Table) of ovarian endometrioid carcinoma showing glandular proliferation, with complex architecture and moderate cytological atypia. The corresponding androgen receptor (AR), estrogen receptor (ER), and progesterone receptor (PR) immunostains show similarly diffuse nuclear staining of the malignant cells. (10 × objective).

AR, ER, and PR stains were positive in 100% (8/8) of the patients with an intensity of combined 2 + and 3 + as shown in S1 Table and represented in Fig 4. Overall, the cellular positivity ranged from 1% to 95% (S1 Table). The IHC reactions for the three receptors were positive for all patients resulting in a presumed *p*-value of 1 (Table 2).

Six cases in this category had been tested for MMR, in which the stains showed intact proteins in all 6 cases (S1 Table).

p53 immunostain was performed on five cases which all showed a wild-type reaction pattern (negative) as shown in S1 Table. While all five specimens were positive for the three receptors, they yielded a negative rection for p53. As p53 compared to the receptors, an identical *p*-value of 0.008 was obtained (Table 3).

Among the 8 patients, 50% (4/8) were overweight (BMI ≥ 25) (S1 Table). Comparison of the BMI of ≥ 25 to the receptors, resulted in borderline significant differences (*p*-values = 0.08) (Table 4).

### GCT (granulosa cell tumor)

There were 13 cases in this diagnostic category. Patients’ ages ranged between 18–76 with a median of 61 years (Table 2). Six patients had received chemotherapy among which all had received additional anti-estrogen (anastrozole) treatment. Five of the 6 patients (83%) had recurrence where AR was positive in all 6 cases (Table 5). One patient was lost to follow-up. None of the patients had been treated with anti-androgen agents (S1 Table).

**Fig 5:**
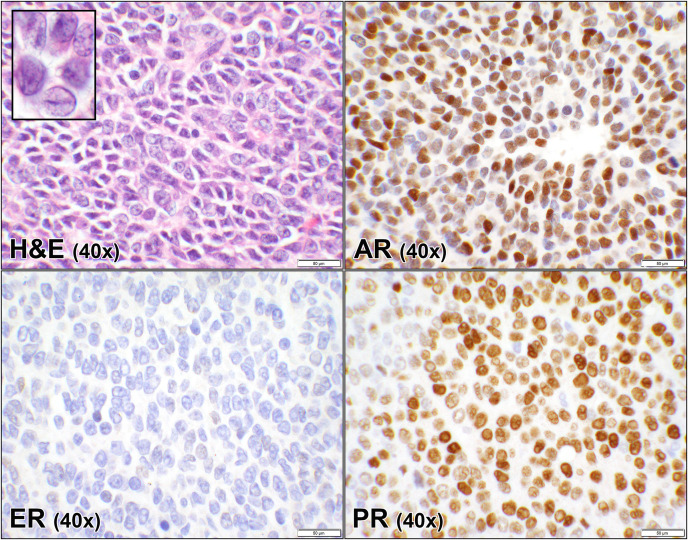
Androgen, estrogen, and progesterone receptors in granulosa cell tumor. Hematoxylin and eosin (H&E) stain of a case (case #62, S1 Table) of ovarian granulosa cell tumor showing diffuse growth pattern of uniform neoplastic cells, with, round to oval nuclei with an irregular nuclear membrane and nuclear grooves (inset), and scant cytoplasm. The corresponding androgen receptor (AR), estrogen receptor (ER), and progesterone receptor (PR) immunostains show strong nuclear staining of the tumor cells. (40 × objective).

AR and PR were positive in 92% (12/13) and ER was positive in 62% (8/13) of the patients as shown in Table 2 and represented in Fig 5. Combined 2 + and 3 + intensities were seen in 15% (2/13) of ER, 84% (11/13) of PR, and 92% (12/13) of PR cases. Maning not only the number of the cases with ER expression was less than those with PR and AR, the intensity of ER expression was also weaker than PR and AR (S1 Table). Overall, the cellular positivity ranged from 10% to 90% (S1 Table). One case showed negative staining with all three hormone receptors. This case was confirmed to be GCT by other expert opinions and also was found to have a FOXL2 mutation. Fisher’s Exact test returned *p*-values of ≥ 0.2 when the stains were compared to each other in this diagnostic category indicating insignificant differences, particularly the *p*-value of PR versus AR was 1 (Table 2).

GCTs were excluded for statistical analyses of p53 since none of the cases had been tested with p53 IHC.

Six patients, 46% (6/13), were overweight (BMI ≥ 25) of whom 15% (2/13) were obese. Comparison of the BMI to the receptors, resulted in insignificant differences (*p*-values ≥0.2) by Fisher’s Exact test (Table 4), indicating concordances between being overweight and the three receptors’ expression.

## Discussion

At the outset, based on the results of this study, it appears that androgen receptor is expressed at high rates and high intensities in the major types of ovarian cancers with the exception of clear cell carcinoma of the ovary supporting its potential as a target for therapy in ovarian cancer. When compared to ER and PR, higher rates of positivity and stronger intensity of the AR expression have been observed among the ovarian malignancies. Even when none of the cases with CCCA has had any positivity for ER or PR, AR has shown expression in one case at the rate of 17% (Table 2). The very low rates of ER and PR expression in CCCA is also has been observed by other investigators [53]. Also of note, AR has been expressed in 100% of the cases with LGSCA and OECA similar to ER and PR (Table 2). In HGSCA and GCT categories, AR expressions were identical at a rate of 92% (Table 2). Overall, AR is positive in 87% of the malignancies combined, while ER and PR are expressed at the rates of 77% and 61% respectively (Table 2). Of note, co-expression of AR, ER, and PR with high intensity staining are seen in 100% of OECAs. This finding is similar, if not identical, to the expression of the receptors in endometrial endometrioid cancers (Table 6) [34].

**Table 6:** Summary of the literature comparing concomitant androgen and estrogen receptors positivity, by immunohistochemistry in multiple studies, in selected ovarian and endometrial cancers.

Authors	Inst	Year	n		LGSCA (AR+)					HGSCA (AR+)				CCCA (AR+)				ECA (AR+)				GCT (AR+)				All (AR+)			
			**Ov**	End	**Ov**			End		**Ov**		End		**Ov**		End		**Ov**		End		**Ov**		End		**Ov**		End	
***Androgen*** Receptor					**n**		**%**	n	**%**	n	**%**	n	**%**	n	**%**	n	**%**	n	**%**	n	**%**	n	**%**	n	**%**	n	**%**	n	**%**
																												
Current Series	UCLA	2025	62		10	**100**		na		23	**92**			1	**17**			8	**100**			12	**92**	na		54	**87**		
Gogola-Mruk et al. [33]	JUPol	2024	15		5	**100**				8	**80**															13	87		
Moh et al. [54]	CCCO	2024	61																			55^*^	**90**			55*	**90**		
Moatamed et al. [34]	UCLA	2023		55				na				12	**92**			3	**50**			35	**97**			na				50	**91**
Summey et al. [55]	MCW	2022	7					na														7	**100**	na		7	**100**		
Cao et al. [56]	HNSM	2022		29				na				7	**88**			1	**9**			6	**67**			na				14	**48**
Elrawi et al. [32]	CUEg	2020	68							31	**55**							4	**33**							35	**51**		
Zadeh et al. [57]	UVC	2018		40				na				7	**70**			3	**30**			11	**65**			na				21	**53**
Tangen et al. [58]	UIB	2016		667				na				36	**55**			7	**25**			386	**67**			na				429	**64**
	**Total** (AR)		213	791	15	**100**				62	**68**	62	**65**	1	**17**	14	**25**	12	**60**	438	**69**	74	**91**			164	**77**	514	**65**
																												
					**LGSCA** (ER+)					**HGSCA** (ER+)				**CCCA** (ER+)				**ECA** (ER+)				**GCT** (ER+)				**All **(ER+)			
					**Ov**			End		**Ov**		End		**Ov**		End		**Ov**		End		**Ov**		End		**Ov**		End	
***Estrogen*** Receptor					**n**	**%**		n	**%**	n	**%**	n	**%**	n	**%**	n	**%**	n	**%**	n	**%**	n	**%**	n	**%**	n	**%**	n	**%**
																												
Current Series	UCLA	2025	62		10	**100**		na		22	**88**			0	**0**			8	**100**			8	**62**	na		48	**77**		
Gogola-Mruk et al. [33]	JUPol	2024	15		5	**100**				7	**70**															12	**80**		
Moatamed et al. [34]	UCLA	2023		55				na				11	**85**			0	**0**			35	**97**			na				46	**84**
Cao et al. [56]	HNSM	2022		29				na				4	**50**			1	**9**			6	**67**			na				11	**38**
Elrawi et al. [32]	CUEg	2020	68							47	**83**							9	**75**							56	**82**		
Zadeh et al. [57]	UVC	2018		40				na				8	**80**			6	**60**			18	**90**			na				32	**80**
	**Total** (ER)		145	124	15	**100**				76	**84**	23	**74**	0	**0**	7	**25**	17	**85**	59	**88**	8	**62**			116	**80**	89	**72**

**Ov**, ovary; **End**, endometrium; **Inst**, institution; **LGSCA**, low grade serous carcinoma; **HGSCA**, high grade serous carcinoma; **CCCA**, clear cell carcinoma; **ECA**, endometrioid carcinoma; **GCT**, granulosa cell tumor; **AR**, androgen receptor; **ER**, estrogen receptor; **+ **, positive; **na**, not applicable; **Endo**, endometrium; **UCLA**, University of California, Los Angeles; J**UPol**, Jagiellonian University, Poland; **CCCO**, Cleveland Clinic, Cleveland, Ohio; **MCW**, Medical College of Wisconsin, Milwaukee, Wisconsin; **HNSM**; Donald and Barbara Zucker School of Medicine at Hofstra/Northwell School of Medicine, Hempstead, New York; **CUEg**, Cairo University, Egypt; **UVC**, University of Virginia, Charlottesville; **UIB**, University of Bergen, Norway. *, indicates the number was extracted from the total based on the stated “>90%” positivity.

Conversely, GCTs have shown co-expression of AR with PR, rather than ER (Table 2). Summey et al. also have observed strong expression of AR in all their cases of GCT [55]. However, since the expression of ER or PR had not been reported, therefore no respective results were available for inclusion in Table 6. These investigators had utilized a novel triple therapy treatment regimen for recurrent adult GCTs in their series. The regimen included a combination of an androgen receptor antagonist, aromatase inhibitor, and a gonadotropin-releasing hormone receptor agonist for hormonal blockade [55]. A majority of those patients (86%) saw clinical benefit and demonstrated measurable responses [55].

As the rate of recurrence (resistance to conventional chemotherapy) is very high [59], regardless of the Fisher’s exact test results in this study (Table 5), nearly all patients with the cancer recurrence exhibited strong AR expression, except for those with CCCA. Therefore, at least the recurrent cancers are potentially eligible for the anti-androgen/androgen receptor-antagonist therapy. In a very recent study, the investigators used ovarian epithelial cells in women at high risk for developing ovarian cancer as well as using the ovaria cancer cell lines, with low-risk patients’ ovarian epithelial cells as control. When they treated the epithelial cells, from the high-risk patients, flutamide (an anti-androgen agent) normalized the cellular miRNA levels to those seen in the cells of low-risk individuals. Flutamide also increased miR-449a and miR-449b-5p levels in cancer cell lines leading to reduction of the AR protein levels and colony stimulating factor 1 receptor, both of which contribute to ovarian cancer progression. Their findings support potential of flutamide as a chemo-preventive agent in ovarian cancers [60].

Unlike the endometrial cancers, there is a paucity or almost non-existence of systematic hormonal receptors’ studies in the literature for ovarian cancers with the exception of two publications [61,62]. Lee et al. have done a systematic study of the hormone receptors on tissue microarray containing different ovarian cancers. Since their tumor classification was based on the old WHO classification [63], without LGSCA and HGSCA distinction, and the hormonal receptors’ expressions were categorized as < 10% and > 10%, therefore their results are not included in Table 6 [61]. de Toledo et al. also performed a similar study of the steroid receptors in ovarian cancers in 2014 [62]. In their study, they had divided the ovarian cancers into serous versus non-serous ovarian tumors with no further subclassifications. Subsequently, their findings could not be included in Table 6. Therefore, our focus on AR will be mostly on our findings for these malignancies based on the current WHO classification [39].

Our results indicate a high rate and a strong expression of AR in both categories of serous carcinomas. This finding is particularly notable among HGSCAs, in which AR expression is seen in 92% of cases, while ER and PR are expressed in 88% and 44% of these cases, respectively (Table 2). Similarly, despite a lack of statistical significance, all LGSCAs have expressed AR with 100% ER and 70% PR expressions (Table 2). Also, both types of serous carcinomas demonstrate similar patterns of ER and AR co-expression, and any significant differences between the three hormone receptors could be attributed to lower rates of PR expression (Table 2). Previous studies have similarly shown high percentages of AR expression in the serous subtype. Independently, Lee et al. and Toledo et al. have found androgen receptor positivity to be higher in serous rather than other tumor types [61,62].

Finally, one of the most notable findings is seen in the clear cell carcinoma (CCCA) category, in which AR is the solely expressed receptor. One of the 6 six cases has demonstrated AR positivity with moderate intensity (2+) despite the lack of ER and PR expression (case #39, S1 Table). There is also a rare case of HGSCA which demonstrated strong AR positivity (3+) without co-expression of ER or PR (case #22, S1 Table). While the number of cases demonstrating this finding is very small, we acknowledge the possibility of a link between AR expression and these higher-grade ovarian malignancies. These malignancies often exhibit high resistance to chemotherapy agents and are associated with high mortality and poor prognosis [64]. This is in contrast to the results from Tangen et al. (2016) showing that AR expression is associated with low grade tumors [58]. Regardless, AR may still be a driver for tumor growth and progression, and therefore, a potential target for the therapy.

In Table 6, we compare our findings with recently published papers that classify AR and ER by different diagnoses in the endometrium. Despite inherent biases and limitations due to different case selection criteria and methodologies, which may have contributed to the variability of hormone receptor expression in all of these studies, the high rates of AR expression are still observed, and in most instances more than ER or PR [34,55–58]. Additionally, AR expression is more prevalent in the ovary than the endometrium, although this trend is also seen with ER expression (Table 6).

Our findings show an overall lack of concordance between p53 and BMI of ≥ 25 with the three hormone receptors (Table 3 and Table 4). However, there were a few correlations among the specific diagnostic categories, none of which reached a statistical significance (*P*-values >0.05). Further studies using a larger sample size and a randomized uniform patient population may elucidate the relationship between AR and these biomarkers or the clinical factors.

Therapeutic strategies targeting ER and PR, especially when expressed by cancer cells, are effective forms of treatment in various gynecologic malignancies [65]. In addition, there is a long history of experience using anti-androgen/AR agents in male prostate and female breast cancers, making it an appealing target to investigate in other hormone driven cancers. Although it has been hypothesized that AR-positive ovarian tumors may also preferentially respond to treatment with AR antagonists, it remains to be determined whether AR status could serve as a reliable prognostic biomarker in this disease model. Experimental, preclinical studies have shown increased cell proliferation and division of AR-expressing epithelial ovarian cancer cells in tissue culture with androgenic stimulation and the opposite effect with androgenic inhibition [10,66]. In contrast, no significant results have been reported from clinical trials, with initial studies showing limited benefit of AR blockade in ovarian cancer except for a subset of these patients who benefited from androgen deprivation therapy [12–15]. A recent phase II trial looked at the use of enzalutamide as a potential treatment option in cases of AR-expressing low- and high-grade serous ovarian cancers [67]. Enzalutamide is a second-generation anti-androgen which inhibits several steps of AR activation leading to cellular apoptosis [67]. They found enzalutamide to confer modest progression-free survival benefit with minimal toxicity in those patients [67]. Mizushima et al., in their fairly recent review article, have concluded that AR inactivation holds promise not only as a potential therapeutic strategy for ovarian cancer but also as a method for enhancing chemosensitivity, especially in patients with AR-positive tumors [68]. Additionally, a review article by Chung et al. has summarized the effect of androgen/AR inhibitors by different investigators in ovarian cancers [2]. The responds ranged from failure [14], to efficacy in response [66], and suppressing ovarian serous carcinoma [11].

Since the long-term therapeutic effects of enzalutamide in ovarian cancer is unknown, future studies are warranted, also investigating this drug in combination with other hormonal agents. It is crucial to keenly consider paradoxical promotion of the disease progression while on anti-androgen therapy, as in prostate cancer [24], in ongoing and future anti-androgen therapies of gynecological cancers. At the time this study concluded, there were no ongoing clinical trials using AR targeted therapy in patients with ovarian cancer that we were aware of.

Strengths of our study include the unique classification and comparison of AR, ER, and PR in each diagnostic category of ovarian cancer. Our findings appear to be in line with the previous studies proposing AR as a potential therapeutic target in hormone-associated gynecologic malignancies [34,55,67]. Limitations of our work include the small sample size, particularly the few numbers of cases in each diagnostic category, and retrospective nature of the study. This study was also subject to selection bias since 62 out of 399 cases were preselected based on whether ER and PR testing was already performed during the course of patient care. While we found certain tumors to show strong or high rates of AR expression by immunohistochemical staining, the functionality of this hormone receptor in the ovarian cancers remains unknown; we have yet to determine if increased androgen exposure is a driver in tumor progression.

### Conclusions

In summary, AR is found to be expressed in a high percentage of most ovarian malignancies with higher rates of positivity and stronger intensity than ER and PR, similar to the study on endometrial cancers [34]. Notably, a significant expression of AR was seen in cases of high-grade serous carcinoma and clear cell carcinoma in the absence of ER and PR expression. We cautiously propose the evaluation of AR expression in ovarian cancer, particularly in specific tumor types that may lack expression of other hormone receptors or biomarkers. As the relationship between AR and treatment response to anti-androgen/AR-antagonist therapies in ovarian cancers is still unknown and worthy of further investigation. Larger prospective studies and clinical trials are necessary to establish the therapeutic effects of the anti-androgen/anti-AR agents on ovarian cancers.

## Supporting information

S1 Table Summary of the patients’ data. (PDF)

S1 Fig Sample pictures of the control tissues and the AR IHC stain. (TIF)
