# Supplementary material for: Significance of androgen receptor and its potential for anti-androgen/androgen receptor-antagonist therapy in ovarian cancers
Source: PLoS One. 2025 May 20;20(5):e0322744. doi: 10.1371/journal.pone.0322744 (PMC12091818; doi:10.1371/journal.pone.0322744)
Supplement: S1 Table — (PDF) [file pone.0322744.s001.pdf]

S1 Table. Summary of the demographic information, diagnostic characteristics, staging, BMI, therapy status, recurrence, and immunohistochemical reactions of the three receptors and p53 in the patients with ovarian cancers

No.	Diagnosis	FIGO	ER		PR		AR		Other Tests	p53	BMI	Stage	Hrmn Rx	Chemo Rx	RR	V-Status
			Cells %	Intensity	Cells %	Intensity	Cells %	Intensity	Status	Status						
1	LGSCA		90%	3+	1%	1+	100%	3+	NP	WT	21.88	pT3cN0	AI	C+P	56.9	90 §
2	LGSCA		90%	3+	70%	3+	99%	3+	NP	WT	29.37	pT1aNo	NF	NF	NF	80 §
3	LGSCA		80%	3+	10%	2+	60%	2+	NP	WT	20.52	pT3cN0	NF	NF	NF	64 §
4	LGSCA		100%	3+	Neg		100%	3+	NP	WT	16.65	pT3cN0	No	C+P	10.7	63
5	LGSCA		99%	3+	25%	2+	99%	3+	NP	WT	24.23	pT1aNo	NF	NF	NF	42 §
6	LGSCA		95%	2+	Neg		60%	2+	NP	WT	24.37	pT3cN1	No	C+P	16.9	23
7	LGSCA		90%	3+	20%	2+	90%	3+	NP	WT	36.49	pT3aNx	AI	C+P	No	29 §
8	LGSCA		90%	3+	Neg		30%	2+	NP	WT	20.23	pT1aNo	No	C+P	76.9	102 §
9	LGSCA		95%	2+	95%	2+	95%	2+	NP	WT	22.13	pT1aNo	No	No	No	18 §
10	LGSCA		95%	2+	5%	2+	95%	2+	NP	WT	38.71	pT3bNx	AI	C+P	0.6	17 §
11	HGSCA		80%	3+	30%	3+	70%	3+	NP	AE-P	21.00	pT3N0	No	C+P	7.3	9
12	HGSCA		Neg		Neg		Neg		NP	AE-P	22.38	pT2N0	No	C+P	No	29 §
13	HGSCA		90%	3+	5%	1+	80%	2+	Her2-Neg	AE-P	31.37	ypT3Nx	No	C+P	21.5	26 §
14	HGSCA		60%	3+	80%	3+	90%	3+	NP	AE-P	28.07	pT1cNx	No	C+P	No	26 §
15	HGSCA		70%	2+	60%	3+	60%	3+	NP	AE-N	23.81	pT1bN0	No	C+P	No	26 §
16	HGSCA		90%	3+	30%	3+	90%	3+	NP	AE-P	20.63	pT3aN0	No	C+P	No	25 §
17	HGSCA		60%	2+	60%	3+	95%	3+	Her2-Neg	AE-N	31.00	pT1bN0	No	C+P	No	25 §
18	HGSCA		30%	3+	Neg		70%	3+	NP	AE-P	22.96	ypT2bNx	No	C+P	30.3	24 §
19	HGSCA		80%	3+	2%	1+	40%	3+	NP	AE-P	28.15	pT3cNx	No	C+P	31.9	24 §
20	HGSCA		80%	3+	Neg		90%	3+	NP	AE-P	27.00	ypT3N1b	No	C+P	3.1	21
21	HGSCA		Neg		Neg		Neg		NP	AE-N	24.00	ypT3Nx	No	C+P	11.6	12
22	HGSCA		Neg		Neg		80%	3+	NP	AE-P	32.01	ypT2N0	No	C+P	27.8	21 §
23	HGSCA		80%	2+	95%	3+	90%	3+	NP	AE-P	27.00	ypT3N0	No	C+P	No	19 §
24	HGSCA		90%	3+	Neg		90%	2+	NP	AE-P	23.25	pT3cNx	No	C+P	No	19 §
25	HGSCA		90%	3+	5%	3+	20%	3+	NP	AE-P	29.27	pT3cN1	No	C+P	12.6	19 §
26	HGSCA		80%	2+	10%	2+	80%	2+	NP	AE-P	23.21	pT3bNx	No	C+P	No	19 §
27	HGSCA		90%	2+	Neg		90%	3+	NP	AE-P	17.96	pT3cN1b	No	C+P	8.7	18 §
28	HGSCA		90%	2+	Neg		90%	3+	NP	AE-P	27.41	pT3cNx	No	C+P	12.7	16 §
29	HGSCA		20%	1+	Neg		20%	2+	NP	AE-P	24.42	pT1aNo	NF	NF	NF	16 §
30	HGSCA		30%	2+	5%	1+	5%	1+	NP	AE-P	23.71	pT3cNx	No	C+P	No	15 §
31	HGSCA		40%	1+	Neg		60%	2+	NP	AE-P	21.51	pT3bN1a	No	C+P	13.1	14 §
32	HGSCA		5%	1+	Neg		10%	2+	NP	AE-P	19.34	pT3aN0	No	C+P	1.0	11 §
33	HGSCA		70%	1+	Neg		80%	2+	NP	AE-P	20.52	pT3N0	NF	NF	NF	11 §
34	HGSCA		75%	2+	Neg		90%	3+	NP	AE-P	26.24	pT2bN0	No	C+P	No	10 §
35	HGSCA		25%	1+	Neg		25%	1+	NP	AE-P	20.14	pT3N0	No	C+P	No	10 §
36	CCCA		Neg		Neg		Neg		NP	WT	22.91	pT3N1	No	C+P	13.6	64 §
37	CCCA		Neg		Neg		Neg		NP	WT	21.11	pT1cN0	No	C+P	No	52 §
38	CCCA		Neg		Neg		Neg		NP	WT	29.72	pT3Nx	No	C+P	5.1	10
39	CCCA		Neg		Neg		20%	2+	MMR-Int	NP	27.34	pT1aN0	No	C+P	No	32 §
40	CCCA		Neg		Neg		Neg		NP	WT	17.64	pT3N1b	No	C+P	17.5	24 §
41†	CCCA		Neg		Neg		Neg		Her2-Neg	NP	27.00	pT3Nx	NF	NF	NF	25 §
42	OECA	1	70%	2+	10%	2+	70%	2+	NP	WT	26.53	pT2bN0	No	C+P	No	92 §
43	OECA	1	100%	3+	100%	3+	20%	2+	MMR-Int	WT	28.50	pT3aNx	No	C+P	56.2	68 §
44	OECA	1	70%	2+	80%	2+	70%	2+	MMR-Int	NP	18.77	pT1aNo	No	No	No	54 §
45	OECA	1	85%	2+	90%	3+	20%	3+	MMR-Int	NP	27.97	pT1aNo	No	C+P	No	48 §
46	OECA	1	70%	3+	10%	3+	70%	3+	MMR-Int	NP	21.14	pT1aNo	No	No	No	24 §
47	OECA	1	90%	3+	90%	3+	90%	3+	NP	WT	24.37	pT1aNo	No	No	No	22 §
48	OECA	2	90%	3+	90%	3+	90%	3+	MMR-Int	WT	24.28	pT2CNx	NF	NF	NF	51 §
49	OECA	2	95%	3+	80%	3+	30%	3+	MMR-Int	WT	28.18	pT1aNx	No	No	No	43 §
50	GCT		Neg		70%	2+	80%	3+	NP	NP	29.29	pT1aN0	No	No	No	89 §
51	GCT		50%	1+	70%	2+	100%	3+	NP	NP	22.12	PT3aN0	AI	G+C	28.3	88 §
52	GCT		60%	1+	75%	3+	100%	3+	NP	NP	24.30	pT1aN0	No	No	No	77 §
53	GCT		90%	2+	90%	3+	95%	3+	NP	NP	27.00		NF	NF	NF	72 §
54	GCT		Neg		40%	1+	100%	3+	NP	NP	22.96	pT1aNx	No	No	No	69 §
55	GCT		25%	1+	80%	2+	90%	3+	NP	NP	32.52	pT1aN0	AI	C+P	11.6	62 §
56	GCT		90%	1+	90%	3+	100%	3+	NP	NP	27.00	pT1aNx	AI	C+P	83.4	139 §
57	GCT		30%	1+	90%	3+	100%	3+	NP	NP	33.27	pT2bNx	AI	C+P	No	36 §
58	GCT		Neg		75%	3+	90%	3+	NP	NP	26.25	pT1aNx	No	No	No	21 §
59	GCT		70%	2+	70%	2+	70%	2+	NP	NP	22.00	pT1aNx	AI	C+P	95.7	115 §
60	GCT		75%	1+	90%	3+	95%	3+	NP	NP	29.37	pT1aNx	AI	C+P	120.3	139 §
61	GCT		Neg		50%	2+	90%	3+	NP	NP	24.25	pT1aN0	No	No	No	17 §
62	GCT		Neg		Neg		Neg		FOXL2-Mut	NP	28.29	pT1aNx	No	No	No	16 §

NP, not performed; **ER**, estrogen receptor; **PR**, progesterone receptor; **AR**, androgen receptor; **LGSCA**, low grade serous carcinoma; **HGSCA**, high grade serous carcinoma; **CCCA**, clear cell carcinoma; **41†**, metastatic; **OEC**, ovarian endometrioid carcinoma; **GCT**, granulosa cell tumor; **Her2-Neg**, Her2-Neu negative; **MMR-Int**, mismatch-repair-protein intact; **FOXL2-Mut**, FOXL2 gene mutated; **WT**, wild type; **AE**, aberrant expression (**P** = over-expressed, **N** = Null); **NP**, not performed; **BMI**, body mass index (kg/m2), **Red** = obese, **Orange** = over-weight, **Blue** = under-weight; **Hrmn RX**, hormonal treatment; **AI**, aromatase inhibitor; **NF**, no follow-ups; **Chemo RX**, chemotherapy, **C+P**: Carboplatin & paclitaxel, **G+C**: gemcitabine & cisplatin; **RR**, recurrence, months after diagnosis or No recurrence; **V-status**, vital-status, months alive after the initial diagnosis (in Red, have expired), **§**, still alive ~~as of 2023-11-27.~~
