# Supplementary figures and images for: Significance of androgen receptor and its potential for anti-androgen/androgen receptor-antagonist therapy in ovarian cancers
Source: PLoS One. 2025 May 20;20(5):e0322744. doi: 10.1371/journal.pone.0322744 (PMC12091818; doi:10.1371/journal.pone.0322744)

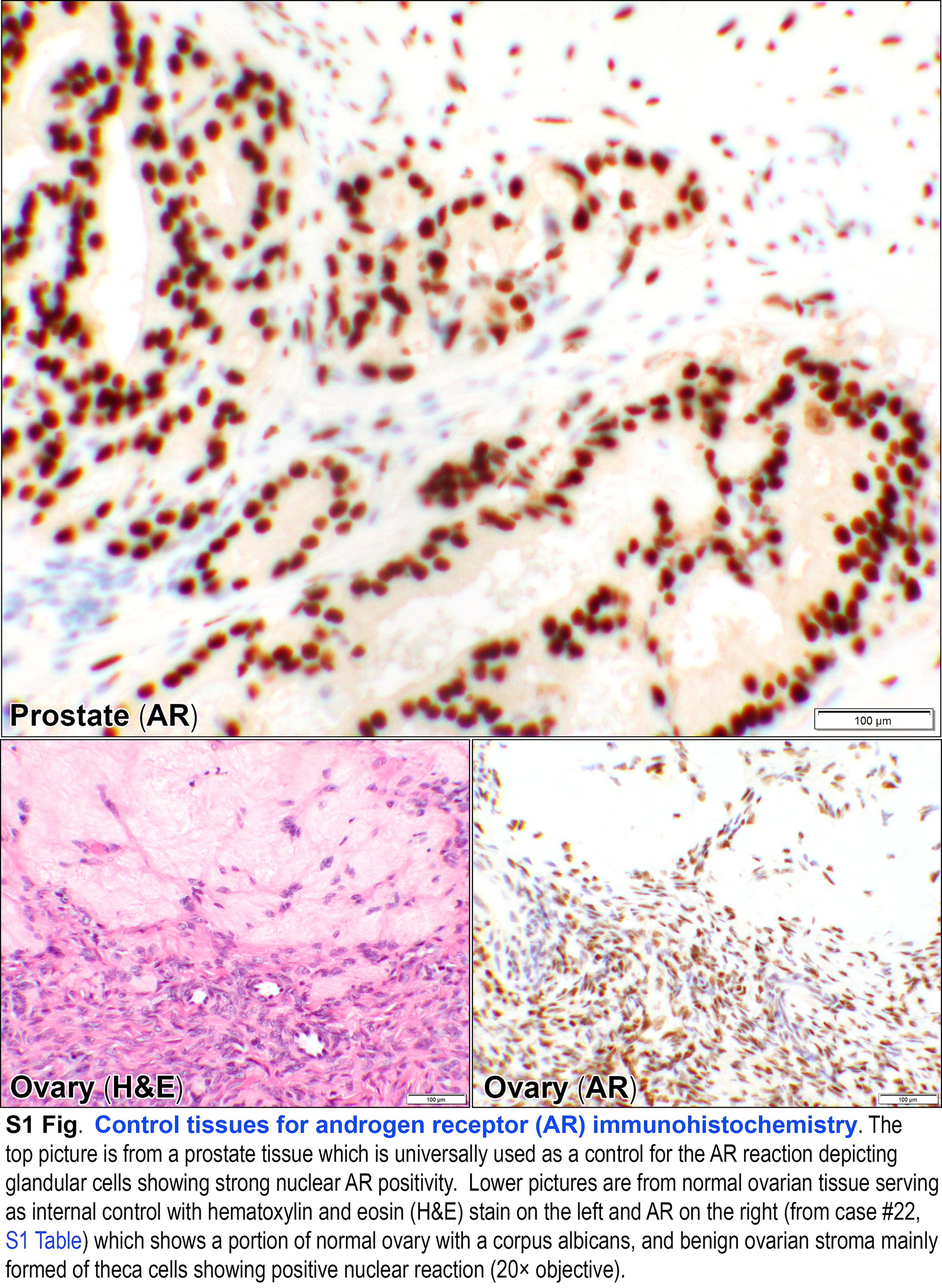

Supplement: S1 Fig — (TIF) [file pone.0322744.s002.tif]
